# Lactate dehydrogenase A inhibition by small molecular entities: steps in the right direction

**DOI:** 10.18632/oncoscience.519

**Published:** 2020-09-09

**Authors:** Btissame El Hassouni, Marika Franczak, Mjriam Capula, Christian M. Vonk, Valentina M. Gomez, Ryszard T. Smolenski, Carlotta Granchi, Godefridus J. Peters, Filippo Minutolo, Elisa Giovannetti

**Affiliations:** ^1^Department of Medical Oncology, Amsterdam UMC, VU University Medical Center, Amsterdam, Netherlands; ^2^Department of Biochemistry, Medical University of Gdansk, Gdansk, Poland; ^3^Fondazione Pisana per la Scienza, Pisa, Italy; ^4^Dipartimento di Farmacia, Università di Pisa, Pisa, Italy

**Keywords:** LDH-A, NHI-Glc-2, warburg, glycolysis

## Abstract

Direct targeting of energy metabolism to defeat cancer is not a recent strategy. Although quite a few drugs use cellular metabolism for their antitumor effect, no direct inhibitors of energy metabolism have been approved by the FDA. Currently, several inhibitors of lactate dehydrogenase A (LDH-A), a key player in glycolysis, are in development. Earlier, we demonstrated the efficacy of *N*-hydroxyindole-based LDH-A inhibitors in different cancer types. In this study we describe the efficacy of NHI-Glc-2, which is designed to dual target cancer cells, by exploiting a simultaneous enhanced glucose uptake by overexpressed glucose transporter 1 (GLUT1) and by inhibition of LDH-A. NHI-Glc-2 inhibits LDH-A enzyme activity, PANC-1 cell growth and disrupts spheroid integrity, with an overall effect that is more pronounced when combined with gemcitabine.

## INTRODUCTION

The high proliferation rate of cancer cells requires sufficient supply of nutrients to enable cell growth [1]. In various cancer types, one of the pathways that is upregulated to provide this increased nutrient demand, is glucose metabolism, i.e. glycolysis. The glucose transporter 1 (GLUT1, also known as solute carrier family 2, facilitated glucose transporter member 1, encoded by the SLC2A1 gene), mediates glucose uptake into the cell, where it is converted via multiple steps to lactate [2, 3]. The last essential step in this pathway is lactate dehydrogenase A (LDH-A), which catalyzes the conversion of pyruvate to lactate using nicotinamide adenine dinucleotide (NADH) as a cofactor. The overexpression of LDH-A contributes to an increased production of lactate, which causes a decrease in the pH of the tumour microenvironment. Both acidification and extracellular accumulation of lactate, contribute to the suppression of immune effectors [4], cause antioxidant defenses against chemotherapeutics [5] and favour tumour invasion [6].

Targeting cancer cells by LDH-A inhibition is, therefore, being explored in various cancer types. A recent study reported that LDH-A inhibition combined with interleukin (IL)-2 led to an increase of stem cell memory T cells (T_scm_), which resulted in an improved anti-tumour response and host survival [7]. In our previous studies, we reported an increased sensitivity to *N*-hydroxyindole-based LDH-A inhibitors, NHI-I and NHI-2, in pancreatic cancer cells growing under hypoxic conditions [8]. Furthermore, the combination of NHI-I or NHI-2 with gemcitabine was synergistic, and led to inhibition of cell migration and invasion. In hypoxic mesothelioma cells, with a low proton-coupled folate transporter expression, an increase of LDH-A was found [9]; hence, treatment with the glucose-conjugated analogue NHI-Glc-2 was examined [10]. Moreover, in these hypoxic mesothelioma models, the combination of NHI-Glc-2 with gemcitabine was synergistic, which supports the hypothesis that LDH-A is a promising target.

In this research perspective we highlight LDH-A inhibition as a strategy to target cancer by showing the effect of hypoxia on LDH-A expression, its inhibition by glucose conjugated inhibitor NHI-Glc-2 in PANC-1 cells and the effects of this compound on spheroids integrity.

**Figure 1:**
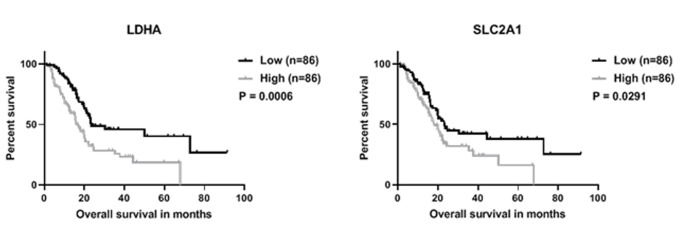
LDH-A and SLC2A1 overexpression correlate with a poor overall survival (OS). Kaplan-Meier curves and log-rank test indicate that a high expression of LDH-A (gray line) correlated with a significantly shorter OS (p-value 0.0006) (left). Similarly, a high expression of SLC2A1 (GLUT1) (gray line) correlated with a lower OS (p-value 0.0291) (right). Groups were separated based on median expression and data was downloaded from LinkedOmics.org and log2 transformed.

## RESULTS AND DISCUSSION

LDH-A is overexpressed in various types of cancer [11–13], and contributes to the aberrant metabolism that stimulates cancer growth and invasion. In a cohort of 172 patients, downloaded from LinkedOmics.org, patients were divided in a low (n=86) or high (n=86) expression group based on the median LDH-A expression (Figure 1, left). In this cohort a high LDH-A expression correlated with a significantly shorter overall survival (OS) (p-value 0.0006), which is in line with previous studies in a cohort of 72 pancreatic cancer patients [13], as well as in different tumour types [12, 14, 15]. In the same dataset, a high expression of SLC2A1 (GLUT1) similarly correlated with a shorter OS (Figure 1, right). Likewise, in a meta-analysis of eight studies comprising 538 cases, a high GLUT-1 expression predicted a shorter OS in patients with pancreatic cancer [16].

Targeting metabolism for cancer treatment to overcome drug resistance is being investigated intensively over years. However, many inhibitors targeting enzymes of the glycolytic pathway did not progress to clinical trials. The few inhibitors that reached phase I trials often failed because of toxicity or lack of efficacy, as summarized previously [2]; therefore more research is warranted. An increased knowledge of the anticancer activities displayed by these compounds does, however, serve as a basis to improve available entities or as a tool for the development of new structures.

**Figure 2:**
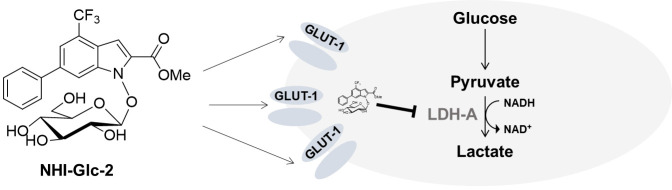
NHI-Glc-2 potential mode of action. Chemical structure of LDH-A inhibitor NHI-Glc-2 (left) and its putative mechanism of action exploiting the overexpression of the glucose transporter 1 (GLUT1, gene SLC2A1) leading to an increased intracellular concentration that can inhibit in cancer overexpressed LDH-A (right) [10].

In this research perspective we show the effect of a structurally modified analogue of NHI-2: NHI-Glc-2. Based on the overexpression of GLUT-1 in pancreatic cancer, NHI-2 was conjugated with a glucose moiety to result in an increased intracellular accumulation [10] (Figure 2). Exploiting both the overexpression of GLUT-1 and LDH-A, this compound is expected to lead to an improved drug response.

**Figure 3:**
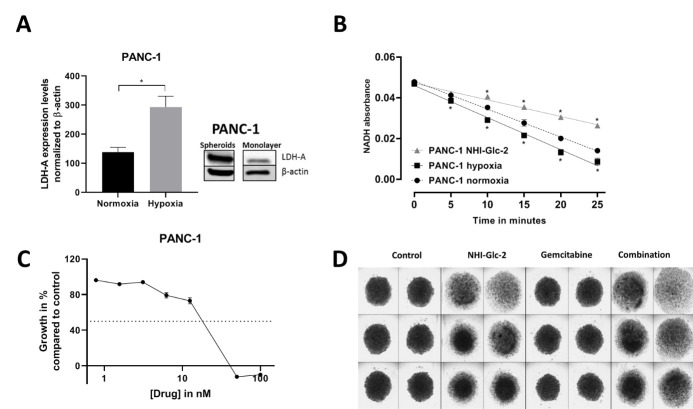
Targeting overexpressed LDH-A in PANC-1 cells. LDH-A mRNA and protein expression is increased under hypoxia (paired t-test p<0.05) and in spheroids, respectively (A). Moreover, LDH-A activity is increased in hypoxia (as indicated by a faster decrease in NADH absorbance) and inhibited by 10 µM NHI-Glc-2 compared to cells grown in normoxia (B). Representative growth inhibition curve of cell growth, error bars indicate standard error of the mean (SEM), showing that NHI-Glc-2 is capable of inhibiting PANC-1 cell growth, with an inhibitory concentration of 50% cell growth (IC_50_) of 14.1 ± 2.7 µM (mean of three experiments performed in triplicates ± SEM) (C). NHI-Glc-2 disrupts spheroid integrity as monotherapy and the effect is more pronounced when combined with gemcitabine (D).

In the tested cell line, PANC-1, an increased LDH-A mRNA expression was observed after growing the cells in a hypoxic environment (1% O_2_) (Figure 3A), supporting previous results [8] and the well-known notion that cells in hypoxia switch to an anaerobic glycolysis. Moreover, cells growing as three-dimensional (3D) spheroids, which possess a hypoxic core [9], had a higher LDH-A protein expression. Besides the overexpression of both LDH-A mRNA and protein, the activity of LDH-A was measured by following the conversion of NADH to NAD+ spectrophotometrically, in which a decrease in NADH indicates a higher LDH-A activity (Figure 3B). PANC-1 cells grown in hypoxia showed the highest LDH-A enzyme activity compared to cells grown in normoxia. Addition of 10 µM of NHI-Glc-2 under normoxic conditions significantly inhibited LDH-A enzyme activity.

NHI-Glc-2 is capable of inhibiting PANC-1 cell growth in the micromolar range (Figure 3C) while most importantly, in a 3D spheroid model assay, treatment with NHI-Glc-2 disrupted spheroid integrity (Figure 3D). However, the commonly used anticancer drug gemcitabine does not have any effect in this model, but the combination of gemcitabine and NHI-Glc-2 had a more pronounced effect on the spheroids compared to either monotherapies. Nonetheless, more research in a variety of cell lines and models is warranted, both under normoxia and hypoxia.

## CONCLUSION

The pivotal role of LDH-A in cancer cell metabolism, its overexpression and, most importantly, its correlation with a poor overall survival when overexpressed, make LDH-A a promising therapeutic target. The structurally optimized LDH-A inhibitor NHI-Glc-2 showed efficacy in mesothelioma models [9]. A similar antitumour activity was observed in pancreatic cancer cells, including gemcitabine-resistant 3D models, which better mimic both cell–cell dynamics in tumour-like spatial structures and low oxygen tension in hypoxic cores. These findings are promising and should prompt further research on NHI-Glc-2, with a special focus on chemoresistant tumours, which rely on metabolic aberrations fuelling anabolic processes, such as pancreatic cancer [17].

## MATERIALS AND METHODS

### LDH-A correlation with overall survival

Correlation of LDH-A and SLC2A1 expression to overall survival (OS) was performed on log2 transformed data downloaded from LinkedOmics [18]. Furthermore, the Kaplan Meier curves were computed in Graphpad (version 8.2.1) using a median cut-off and log-rank test for significance.

### Cell culture

PANC-1 (ATCC® CRL-1469) cells were grown in RPMI medium (Gibco, USA) supplemented with 10% fetal bovine serum (Biowest, France) and 1% penicillin-streptomycin (Lonza, Switzerland) and were maintained at 37⁰C, 5% CO_2_.

### RT-qPCR

Gene expression of LDH-A under normal and hypoxic (72hours, 1% O_2_) conditions was assessed by RT-qPCR as described previously [8].

### Western blot

Samples containing 50 µg protein of lysed spheroids (day 6) and cells grown in monolayer were separated on Mini-PROTEAN® TGX^TM^ Precast gels (Bio-rad, United States) as described previously [19]. Antibodies used for target detection; LDH-A (47010, 1:1000, Abcam, United Kingdom), β-actin (4790, 1:1000, Cell signalling, United States), Secondary IRDye® 800CW (926-32230, 1:10 000, Li-COR Bioscience, United States).

### Enzyme activity 

Enzyme activity of LDH-A was measured spectrophotometrically at an absorbance of 340 nm based on the decrease of NADH as described previously [8]. LDH-A enzyme activity was measured in lysates of cells that were grown under normoxia or hypoxia and in normoxia lysates containing 10 μM NHI-Glc-2. A faster disappearance of NADH indicates a higher enzyme activity.

### Drug sensitivity to NHI-Glc-2 

The sensitivity of PANC-1 cells to the LDH-A inhibitor NHI-Glc-2 was assessed by the sulforhodamine-B assay as described previously [20]. In short, 3000 cells per well were seeded in a 96 wells plate and after 24 hours of cell attachment, the cells were treated for 72 hours with a concentration range of NHI-Glc-2.

### 3D spheroid model

Spheroids were formed in ultra-repellent plates (7007, Corning, United States) by seeding 10^4^ PANC-1 cells/well. The spheroids were grown for 12 days, with medium refreshing every three days, followed by 72h treatment with NHI-Glc-2 (1 µM) and/or gemcitabine (100 nM) for 72 hours in normoxia (n=6 per condition) and were visualized on a Leica DMI3000B Microscope at 5x magnification.

## References

[1] Warburg O (1925). The metabolism of carcinoma cells.. J Cancer Res.

[2] El Hassouni B, Granchi C, Vallés-Martí A, Supadmanaba IG, Bononi G, Tuccinardi T, Funel N, Jimenez CR, Peters GJ, Giovannetti E, Minutolo F (2020). The dichotomous role of the glycolytic metabolism pathway in cancer metastasis: interplay with the complex tumor microenvironment and novel therapeutic strategies.. Semin Cancer Biol.

[3] Navale AM, Paranjape AN (2016). Glucose transporters: physiological and pathological roles.. Biophys Rev.

[4] Fischer K, Hoffmann P, Voelkl S, Meidenbauer N, Ammer J, Edinger M, Gottfried E, Schwarz S, Rothe G, Hoves S, Renner K, Timischl B, Mackensen A (2007). Inhibitory effect of tumor cell-derived lactic acid on human T cells.. Blood.

[5] Gatenby RA, Gillies RJ (2004). Why do cancers have high aerobic glycolysis?. Nat Rev Cancer.

[6] Swietach P, Vaughan-Jones RD, Harris AL (2007). Regulation of tumor pH and the role of carbonic anhydrase 9.. Cancer Metastasis Rev.

[7] Hermans D, Gautam S, García-Cañaveras JC, Gromer D, Mitra S, Spolski R, Li P, Christensen S, Nguyen R, Lin JX, Oh J, Du N, Veenbergen S (2020). Lactate dehydrogenase inhibition synergizes with IL-21 to promote CD8
^+^
T cell stemness and antitumor immunity. Proc Natl Acad Sci USA.

[8] Maftouh M, Avan A, Sciarrillo R, Granchi C, Leon LG, Rani R, Funel N, Smid K, Honeywell R, Boggi U, Minutolo F, Peters GJ, Giovannetti E (2014). Synergistic interaction of novel lactate dehydrogenase inhibitors with gemcitabine against pancreatic cancer cells in hypoxia.. Br J Cancer.

[9] Li Petri G, El Hassouni B, Sciarrillo R, Funel N, Mantini G, Zeeuw van der Laan EA, Cascioferro S, Avan A, Zucali PA, Zaffaroni N, Lagerweij T, Parrino B, Smid K (2020). Impact of hypoxia on chemoresistance of mesothelioma mediated by the proton-coupled folate transporter, and preclinical activity of new anti-LDH-A compounds.. Br J Cancer.

[10] Calvaresi EC, Granchi C, Tuccinardi T, Di Bussolo V, Huigens RW, Lee HY, Palchaudhuri R, Macchia M, Martinelli A, Minutolo F, Hergenrother PJ (2013). Dual targeting of the Warburg effect with a glucose-conjugated lactate dehydrogenase inhibitor.. ChemBioChem.

[11] Rong Y, Wu W, Ni X, Kuang T, Jin D, Wang D, Lou W (2013). Lactate dehydrogenase A is overexpressed in pancreatic cancer and promotes the growth of pancreatic cancer cells.. Tumour Biol.

[12] Cai H, Li J, Zhang Y, Liao Y, Zhu Y, Wang C, Hou J (2019). LDHA Promotes Oral Squamous Cell Carcinoma Progression Through Facilitating Glycolysis and Epithelial-Mesenchymal Transition.. Front Oncol.

[13] Mohammad GH, Olde Damink SW, Malago M, Dhar DK, Pereira SP (2016). Pyruvate kinase M2 and lactate dehydrogenase a are overexpressed in pancreatic cancer and correlate with poor outcome.. PLoS One.

[14] Li F, Xiang H, Pang Z, Chen Z, Dai J, Chen S, Xu B, Zhang T (2020). Association between lactate dehydrogenase levels and oncologic outcomes in metastatic prostate cancer: A meta-analysis.. Cancer Med.

[15] Deng F, Chen D, Wei X, Lu S, Luo X, He J, Liu J, Meng T, Yang A, Chen H (2020). Development and validation of a prognostic classifier based on HIF-1 signaling for hepatocellular carcinoma.. Aging (Albany NY).

[16] Sharen G, Peng Y, Cheng H, Liu Y, Shi Y, Zhao J (2017). Prognostic value of GLUT-1 expression in pancreatic cancer: results from 538 patients.. Oncotarget.

[17] Grasso C, Jansen G, Giovannetti E (2017). Drug resistance in pancreatic cancer: impact of altered energy metabolism.. Crit Rev Oncol Hematol.

[18] Vasaikar SV, Straub P, Wang J, Zhang B (2018). LinkedOmics: analyzing multi-omics data within and across 32 cancer types.. Nucleic Acids Res.

[19] El Hassouni B, Mantini G, Immordino B, Peters GJ, Giovannetti E (2019). CX-5461 inhibits pancreatic ductal adenocarcinoma cell growth, migration and induces DNA damage.. Molecules.

[20] Sciarrillo R, Wojtuszkiewicz A, Kooi IE, Gómez VE, Boggi U, Jansen G, Kaspers GJ, Cloos J, Giovannetti E (2016). Using RNA-sequencing to detect novel splice variants related to drug resistance in in vitro cancer models.. J Vis Exp.

