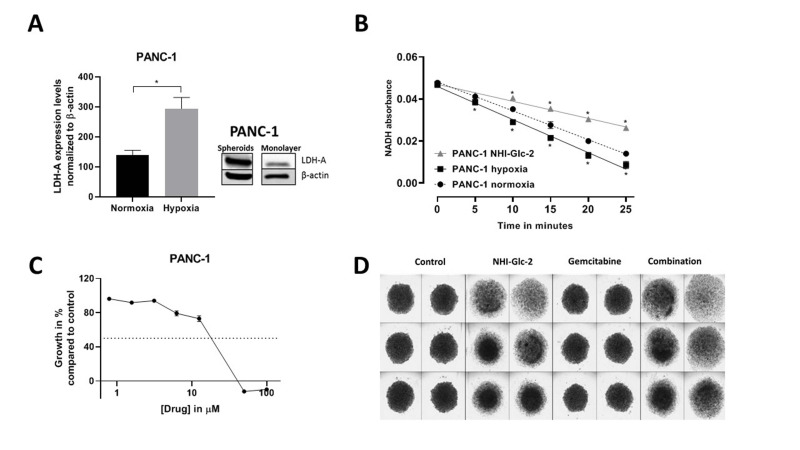# Correction: Lactate dehydrogenase A inhibition by small molecular entities: steps in the right direction

**DOI:** 10.18632/oncoscience.529

**Published:** 2021-03-30

**Authors:** Btissame El Hassouni, Marika Franczak, Mjriam Capula, Christian M. Vonk, Valentina M. Gomez, Ryszard T. Smolenski, Carlotta Granchi, Godefridus J. Peters, Filippo Minutolo, Elisa Giovannetti

**Affiliations:** ^1^Department of Medical Oncology, Amsterdam UMC, VU University Medical Center, Amsterdam, Netherlands; ^2^Department of Biochemistry, Medical University of Gdansk, Gdansk, Poland; ^3^Fondazione Pisana per la Scienza, Pisa, Italy; ^4^Dipartimento di Farmacia, Università di Pisa, Pisa, Italy

Original article: Oncoscience. 2020 Sep 9;7(9-10):76-80

**PMCID**: PMC7640902 **PMID**: 33195739 **DOI**: 10.18632/oncoscience.519

**This article has been corrected**: In Figure 3C, the graph is mislabeled. The correct unit of measurement, µM, is shown in the figure below. The authors declare that these corrections do not change the results or conclusions of this paper.

**Figure 3:**